# Unmasking the Origin of Cutaneous Metastasis: A Case Report

**DOI:** 10.7759/cureus.72156

**Published:** 2024-10-22

**Authors:** Paola Campillo, Adriana Morales Rivera, Alice Kesler, Zein Barakat, Ganga B Ramidi

**Affiliations:** 1 Internal Medicine, Lakeland Regional Health, Lakeland, USA; 2 Infectious Diseases, Lakeland Regional Health, Lakeland, USA

**Keywords:** cutaneous metastasis, histopathology, prognosis, skin metastasis, urothelial carcinoma

## Abstract

Cutaneous metastasis typically represents a late manifestation of internal malignancy. We present the case of a 60-year-old woman with a history of cervical cancer who presented with lower abdominal pain. Imaging studies revealed a high-grade carcinoma involving the urothelium, suggestive of primary urothelial carcinoma. Further investigation uncovered widespread metastatic disease, including cutaneous metastases to the arm and pelvis. Despite a comprehensive diagnostic workup, including immunohistochemical analysis, the primary tumor site could not be definitively identified, underscoring the challenges in diagnosing cutaneous metastases of unknown origin. This case highlights the necessity of considering metastatic disease in patients with atypical skin findings. Moreover, it emphasizes the aggressive nature of cutaneous metastases, particularly those originating from urothelial carcinomas, which are associated with a poor prognosis and limited treatment options.

## Introduction

Cutaneous metastasis originating from internal organ malignancies or soft tissue sarcomas is an uncommon finding, affecting less than 10% of cancer patients [1]. The presentation of skin metastasis can vary, including nodular, inflammatory, and fibrotic types. The most common presentation is the nodular type, which manifests as solitary or multiple rapidly emerging, nonspecific clusters of firm, painless nodules without any clear underlying cause [2,3]. The most frequent metastatic sites for bladder cancer are lymph nodes, liver, lung, bone, and gallbladder [3]. However, the primary carcinomas leading to cutaneous metastases most often include lung cancer, colon cancer, melanoma, and squamous cell carcinoma, particularly of the oral cavity in males, as well as breast cancer, colon cancer, melanoma, and ovarian cancer in females [1]. Interestingly, there is often a significant delay between the diagnosis of the primary malignancy and the recognition of associated skin metastases, sometimes spanning months or even years [4]. Conversely, some cutaneous metastases may serve as the initial manifestation of internal malignancy [5].

Cutaneous metastases can arise through hematogenous or lymphatic spread, direct extension, or surgical implantation of the primary tumor. Cutaneous metastatic disease from urothelial carcinoma is particularly rare, with an incidence estimated at 1.3% for all urological malignancies [3]. Unfortunately, the prognosis for patients diagnosed with cutaneous metastasis from a urologic tumor is very poor, with less than 2% surviving longer than a year and a median disease-specific survival of less than six months [6].

## Case presentation

**Figure 1:**
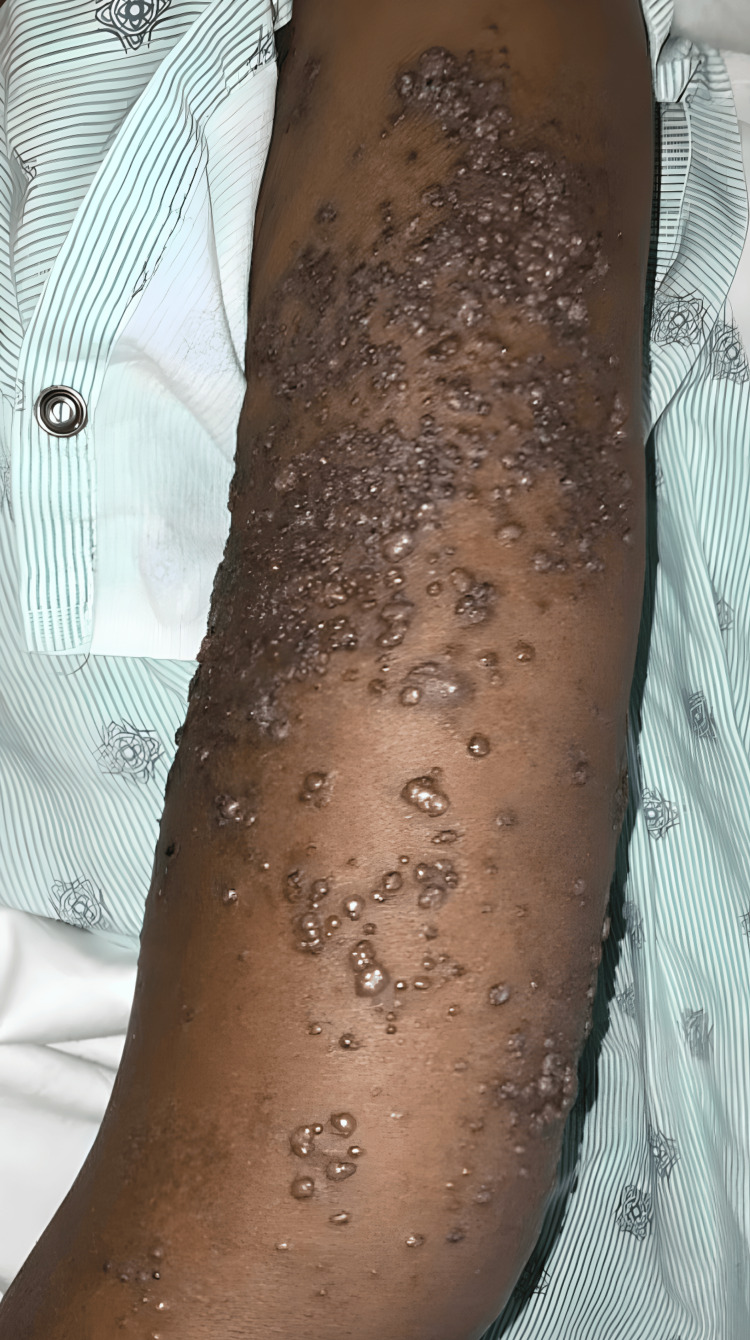
Clinical images (05/2024) showing numerous clusters of firm, painless nodules distributed throughout the upper extremities, bilateral breasts, mons pubis, lower abdomen, and sacrum, along with areas of ulceration

**Figure 2:**
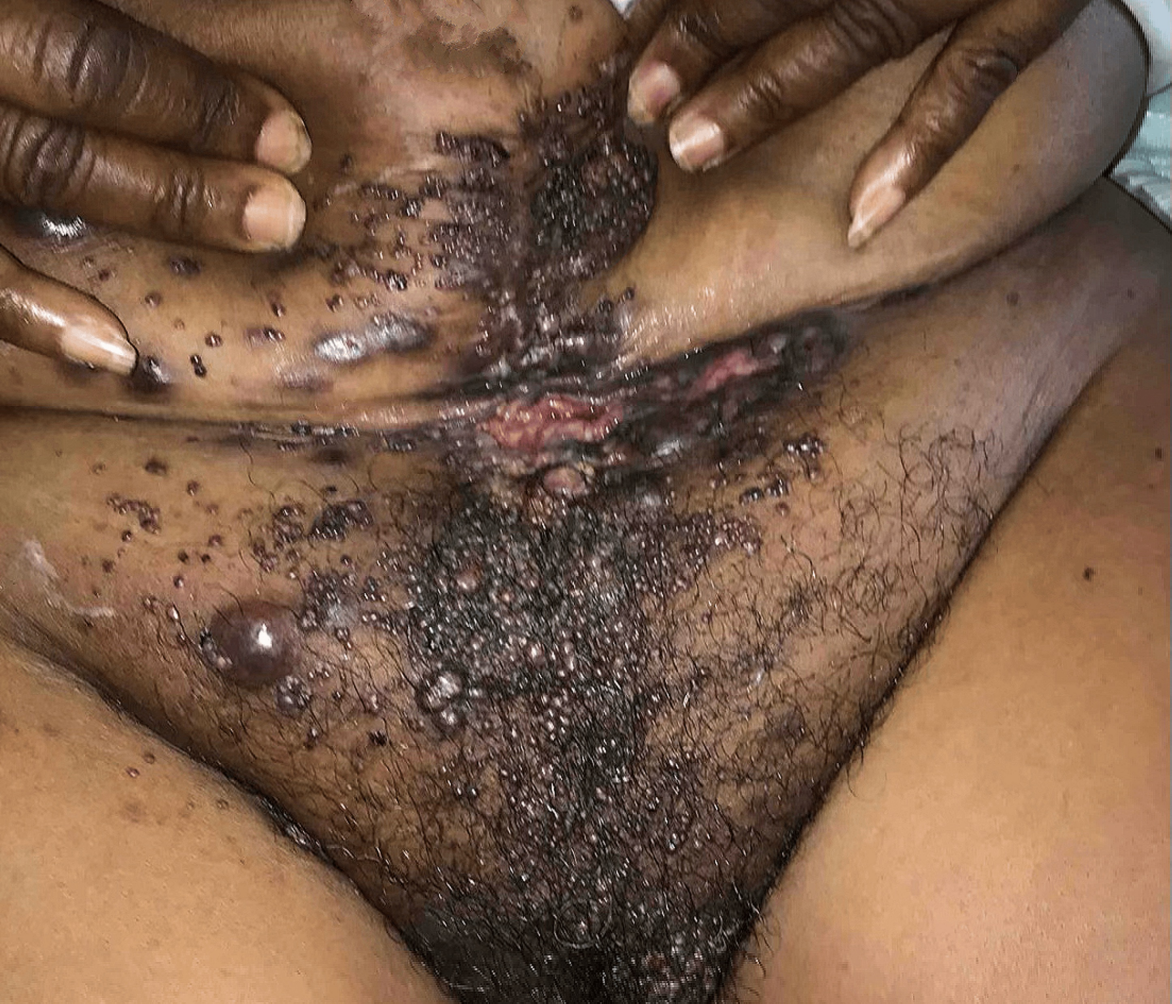
Clinical images (05/2024) showing numerous clusters of firm, painless nodules distributed across the upper extremities, bilateral breasts, mons pubis, lower abdomen, and sacrum, along with areas of ulceration

A 60-year-old female with a history of cervical cancer, status post-hysterectomy, presented with complaints of lower abdominal pain and shortness of breath. During a prior hospitalization, the patient underwent a cystoscopic resection of a tumor at the right orifice. Pathology revealed high-grade carcinoma involving inflamed urothelium and submucosa. Immunohistochemical stains indicated that the immunoprofile was not organ specific, and no in situ urothelial carcinoma component was identified. The findings suggested metastatic carcinoma of unknown origin. Immunohistochemistry (IHC) was positive for CK7 and negative for CK20, CDX2, GATA3, TRPS1, calretinin, WT-1, TTF-1, synaptophysin, chromogranin, p53, CK5/6, SF1, D240, and PAX8, representing a nonspecific organ immunoprofile. During this hospital visit, the patient also complained of “wart-like” lesions on the left arm and in the suprapubic area (Figure 1, Figure 2). A punch biopsy of a left arm lesion revealed metastatic carcinoma with histopathologic findings similar to those of the right ureteral orifice; however, IHC was positive for CK7 and CK20. Since other primary sources could not be entirely excluded, further clinical and radiologic correlation was deemed essential.

**Figure 3:**
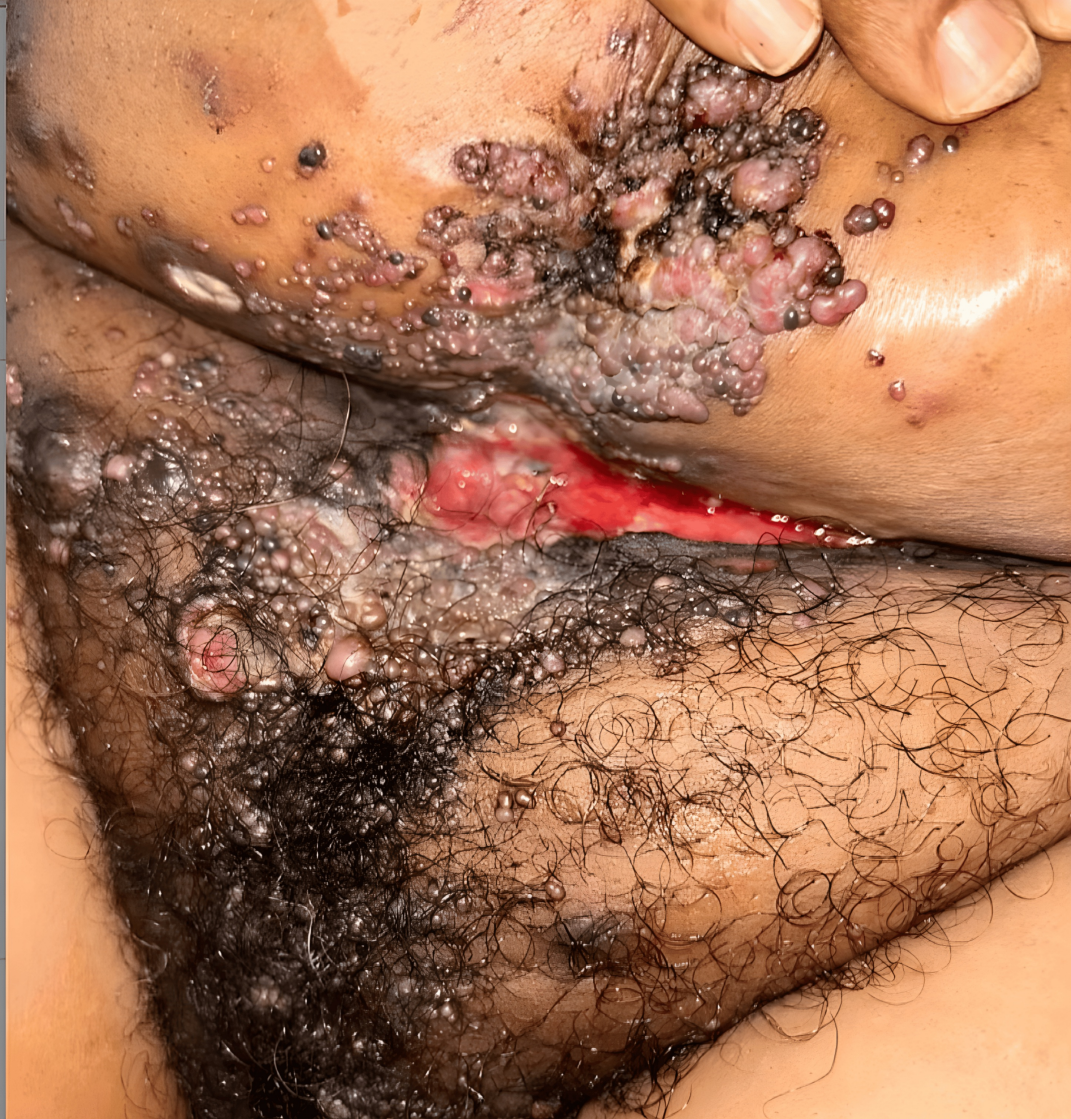
Clinical images (07/2024) showing numerous clusters of firm, painless nodules distributed across the upper extremities, bilateral breasts, mons pubis, lower abdomen, and sacrum, accompanied by areas of ulceration

**Figure 4:**
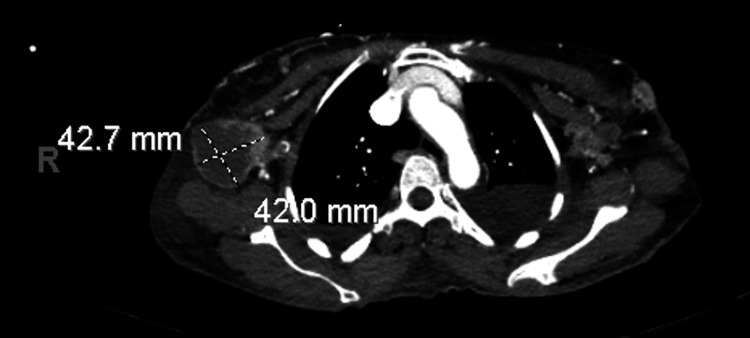
CT scan of the chest with contrast showing a hypovascular mass measuring 42.7 × 42 mm with thickened enhancing walls located within the right axilla, raising concerns for an enlarged, necrotic lymph node

**Figure 5:**
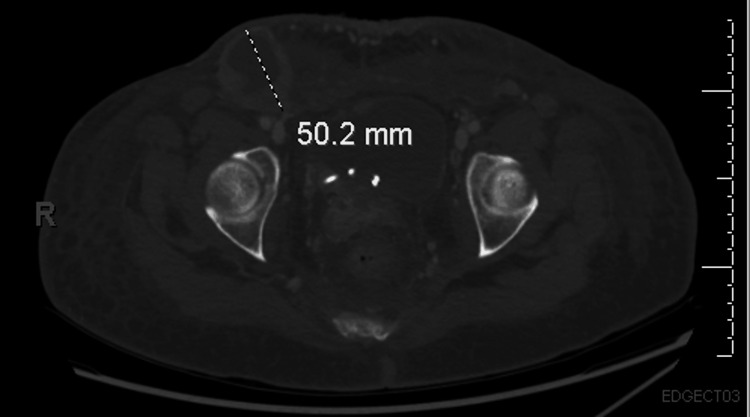
CT scan of the pelvis with contrast revealing a hypovascular lesion measuring 50.2 mm in diameter, characterized by thickened enhancing walls within the right inguinal region, resembling a large necrotic lymph node

Upon presentation at our institution, the patient reported suprapubic and ventral abdominal pain, along with worsening skin lesions that spread to the chest and open wounds in the suprapubic area (Figure 3). Imaging studies, including CT scans of the chest, abdomen, and pelvis, revealed bilateral moderate to large pleural effusions, as well as a 42.7 × 42 mm hypovascular mass within the right axilla, suggestive of a necrotic lymph node (Figure 4). Similar lesions were noted in the pectoralis major muscle on the left and within the breast tissue bilaterally. Mild to moderate dilatation of both renal collecting systems was observed, with the right renal stent out of place. There was no bladder mass or wall thickening, and no retroperitoneal lymphadenopathy was detected. Additionally, a hypovascular mass was identified in the left external iliac chain lymph node, along with similar lesions in the left inguinal lymph node and a 50.2 mm hypovascular lesion in the right inguinal region (Figure 5), consistent with necrotic lymph nodes. Significant edema was noted throughout the soft tissue walls of the chest, abdomen, and pelvis. Pelvic and transvaginal ultrasounds provided nondiagnostic images. Core biopsies of the right inguinal nodes and fine needle aspiration of the right axillary node tested positive for metastatic poorly differentiated squamous cell carcinoma.

The diagnosis of metastatic high-grade carcinoma was confirmed; however, the primary site could not be conclusively identified through histopathologic analysis. Given the advanced nature of the disease, there was no well-defined role for radiation therapy or surgical intervention. The patient was medically stabilized and discharged to continue with palliative care.

## Discussion

While cutaneous metastases are generally infrequent, occurring in 0.7-10% of oncology cases, their appearance often indicates advanced-stage disease and is associated with a poor prognosis, with an average survival of three to five months [1]. This rarity can lead to delays in diagnosis, highlighting the importance of maintaining a high index of suspicion, especially in patients with a history of malignancy who present with suggestive cutaneous findings.

Although cutaneous metastases typically manifest as nodules on the abdomen, it is important to note that most arise locally from the primary site [7]. In our case, however, the patient displayed a more unusual pattern, with metastasis first appearing on the arm and later spreading to the suprapubic area and breasts. While nodules are a common presentation of cutaneous metastasis [8], the distribution of lesions in atypical areas like the arm and breasts complicates the clinical picture, necessitating careful consideration of metastatic disease even in less common locations.

Metastatic lesions can mimic a variety of benign and malignant dermatological conditions, making histopathological confirmation essential in uncertain cases. Hasan et al. demonstrated that cutaneous metastases often resemble primary skin conditions, such as keratoacanthomas and melanomas, further complicating diagnosis [9]. Additionally, a case report described cutaneous metastasis that mimicked erysipelas [10], showcasing the wide spectrum of possible clinical presentations. Given this diagnostic complexity, clinicians must remain vigilant, particularly when confronted with unusual or atypical skin lesions in patients with a known history of malignancy.

In our case, cutaneous metastasis was the first sign of urothelial carcinoma, although the patient only sought medical attention when she began experiencing urinary symptoms. This case illustrates how cutaneous metastasis, despite the typically poor prognosis associated with metastatic disease outcomes [11], can sometimes present before localized symptoms of the primary malignancy. This reinforces the need for early recognition and evaluation of atypical skin lesions, even when they occur prior to other systemic symptoms.

The diagnostic challenge of cutaneous metastases lies in their ability to closely resemble other dermatologic conditions. Histopathology, particularly in challenging cases, requires clinicopathological correlation and immunohistochemical analysis. For example, high-grade transitional cell carcinoma of the urinary tract can be particularly difficult to differentiate from other malignancies. Immunohistochemical markers such as CK7, CK20, and p63, along with coordinated staining patterns, are valuable in distinguishing these malignancies, as noted in the study by Karde et al. [7].

This case, especially considering the patient’s medical history of cervical cancer and the widespread metastatic disease involving the abdominal wall musculature, underscores the aggressive and disseminated nature of the malignancy. Although the primary cancer site was never definitively confirmed, clinical correlation, imaging, and histopathology suggest a likely urothelial origin. Early recognition and accurate diagnosis of metastatic carcinoma are crucial for guiding treatment strategies and potentially improving patient outcomes, despite the poor prognosis typically associated with cutaneous metastasis.

## Conclusions

Cutaneous metastasis from an unknown primary cancer is uncommon and challenging to manage. A comprehensive evaluation is essential for prompt and accurate diagnosis, guiding treatment and potentially improving outcomes, despite the typically poor prognosis. This case underscores the importance of considering metastatic carcinoma when assessing unexplained skin changes.
